# Revisiting the conformational state of albumin conjugated to gold nanoclusters: A self-assembly pathway to giant superstructures unraveled

**DOI:** 10.1371/journal.pone.0218975

**Published:** 2019-06-27

**Authors:** Michał Kluz, Hanna Nieznańska, Robert Dec, Igor Dzięcielewski, Bartosz Niżyński, Grzegorz Ścibisz, Wojciech Puławski, Grzegorz Staszczak, Ewelina Klein, Julita Smalc-Koziorowska, Wojciech Dzwolak

**Affiliations:** 1 Institute of High Pressure Physics, Polish Academy of Sciences, Warsaw, Poland; 2 Nencki Institute of Experimental Biology, Polish Academy of Sciences, Warsaw, Poland; 3 Department of Chemistry, Biological and Chemical Research Centre, University of Warsaw, Warsaw, Poland; Tsinghua University School of Life Sciences, CHINA

## Abstract

Bovine serum albumin (BSA) is often employed as a proteinaceous component for synthesis of luminescent protein-stabilized gold nanoclusters (AuNC): intriguing systems with many potential applications. Typically, the formation of BSA-AuNC conjugate occurs under strongly alkaline conditions. Due to the sheer complexity of intertwined chemical and structural transitions taking place upon BSA-AuNC formation, the state of albumin enveloping AuNCs remains poorly characterized. Here, we study the conformational properties of BSA bound to AuNCs using an array of biophysical tools including vibrational spectroscopy, circular dichroism, fluorescence spectroscopy and trypsin digestion. The alkaline conditions of BSA-AuNC self-assembly appear to be primary responsible for the profound irreversible disruption of tertiary contacts, partial unfolding of native α-helices, hydrolysis of disulfide bonds and the protein becoming vulnerable to trypsin digestion. Further unfolding of BSA-AuNC by guanidinium hydrochloride (GdnHCl) is fully reversible equally in terms of albumin’s secondary structure and conjugate’s luminescent properties. This suggests that binding to AuNCs traps the albumin molecule in a state that is both partly disordered and refractory to irreversible misfolding. Indeed, when BSA-AuNC is subjected to conditions favoring self-association of BSA into amyloid-like fibrils, the buildup of non-native β-sheet conformation is less pronounced than in a control experiment with unmodified BSA. Unexpectedly, BSA-AuNC reveals a tendency to self-assemble into giant twisted superstructures of micrometer lengths detectable with transmission electron microscopy (TEM), a property absent in unmodified BSA. The process is accompanied by ordering of bound AuNCs into elongated streaks and simultaneous decrease in fluorescence intensity. The newly discovered self-association pathway appears to be specifically accessible to protein molecules with a certain restriction on structural dynamics which in the case of BSA-AuNC arises from binding to metal nanoclusters. Our results have been discussed in the context of mechanisms of protein misfolding and applications of BSA-AuNC.

## Introduction

The history of research on small clusters of Au atoms exhibiting strong quantum confinement effects and molecule-like electronic transitions dates back to the 1960s [1]. The field has gained significant momentum in recent years when the full spectrum of potential applications of luminescent AuNCs became clear [2–4] and new facile synthetic strategies were developed [5, 6]. In their surprising work, Xie et al. have shown that at alkaline pH BSA exhibits the whole set of coinciding redox, binding, and macromolecular properties necessary to convert chloroauric (III) acid into AuNCs and stabilize the newly formed clusters through entrapment within the protein envelope [5]. Bare AuNCs are unstable and tend to self-assemble into larger and non-luminescent groupings of Au atoms. In this context, the key role of the protein component becomes clear: it prevents such occurrences by firm covalent attachment (provided by Au-S bonds) of small AuNCs to much larger and singly dispersed albumin partners [2–3, 5–6]. Should AuNCs become untethered from the complex with albumin, as it happens during degradation with proteases, nanoclusters will no longer be protected from self-association and the luminescence will be quenched. The ease of the synthetic route discovered by Xie et al. has galvanized research on various applications of BSA-AuNC conjugates, or of similar systems employing different biomacromolecular components (e.g. [7, 8]), even though the underlying photophysics has not been fully understood [9, 10]. The capacity of serum albumin (bovine or otherwise [11]) to facilitate nucleation and growth of AuNCs and to host them afterwards seems at first rather unsurprising in light of the physiological function of this protein which consists chiefly in transport of numerous endogenous and exogenous compounds including drugs [12, 13]. However, even a moderately alkaline environment destabilizes BSA molecules, as the protein converts into so-called ‘Aged’ form [14]. Strongly alkaline conditions applied during synthesis of BSA-AuNC only further destabilize the native state and perturb BSA’s function as a biomacromolecular ‘carrier’. Hence, binding interactions between AuNCs and BSA are likely to involve a profoundly altered state of the host. Furthermore, on top of strictly conformational changes, the alkaline environment of BSA-AuNC growth may trigger breakage of disulfide bonds [15, 16] and tyrosine dimerization [17]. Should such dramatic perturbations of the covalent structure take place within the AuNCs-binding BSA this would call into question various approaches to study structure and photophysics of this system on the assumption that the albumin envelope remains in a quasi-native state. One tangible aspect of different conformational dynamics of AuNCs-binding albumin vis-à-vis the unmodified protein is the remarkable sensitivity of the former one to enzymatic proteolysis [18], a useful trait for development of luminescent sensors of protease activity.

Several studies have been aimed at characterization of AuNCs-bound state of albumin (e.g. [19–24]) but for a number of reasons including complexity of the system, many fundamental questions remain unanswered. In particular, it is unclear how the binding to AuNCs affects BSA’s capacity to undergo various structural transitions including self-association into amyloid-like fibrils. Aggregation of disordered protein molecules and formation of β-sheet-rich aggregates (amorphous or fibrillar) is a common and clinically important structural transition accessible, in principle, to most, if not all proteins [25, 26]. In vivo, the formation of fibrillar highly ordered aggregates (amyloid fibrils) is often linked to severe degenerative maladies such as Alzheimer’s disease or type II diabetes [27]. On the other hand, the thermodynamic and mechanical properties of amyloid fibrils have made them very promising nanomaterials [28–30]. Since BSA is also capable of self-assembling into amyloid-like fibrils under appropriate conditions [31–32], our initial motivation was to see how this process is affected when unmodified BSA is replaced with BSA-AuNC as a precursor for the self-assembly. As the tendency to aggregate and to form amyloid-like fibrils often coincides with the degree of destabilization of protein’s native structure, biophysical characterization of the conformation and stability of albumin as an integral part of the BSA-AuNC complex became a starting point for this study. We are showing that certain dynamic aspects of the conformational state of BSA-AuNC are unique to this system and affect the course of structural transitions involving the complex. Our findings provide strong evidence that while the presence of bound AuNCs (and covalent modifications caused by the protein-assisted synthesis of AuNCs) appears, at first, to prevent aggregation, it, in fact, forces the protein to step on an entirely different self-association pathway leading to unique giant superstructures.

## Materials and methods

### Materials and preparative procedure

BSA, HAuCl_4_·3H_2_O and other chemicals (all of analytical reagent grade) were purchased from Sigma-Aldrich and were used without further purification. BSA-AuNC complex was synthesized according to a procedure similar to the original synthetic route developed by Xie et al. [5]. Briefly: 500 μl volumes of aqueous solutions of HAuCl_4_ (10 mM) and BSA (50 mg/mL) were mixed vigorously at 37 ^o^C for 2 minutes, after which a 50 μl portion of 1 M aqueous NaOH was added. The thus obtained solution was again mixed and subjected to 12h-long agitation (600 rpm) at 37 ^o^C in an Eppendorf Thermomixer Comfort accessory which resulted in the formation of BSA-AuNC. Samples of alkaline-treated albumin (BSA-Alk) used in control experiments were prepared analogously except that deionized water was used instead of HAuCl_4_ solution. It is important to consider the implications of the BSA:HAuCl_4_ mixing molar ratio at the beginning of synthesis of BSA-AuNC which is approximately 1:13. The initial reaction phases during the synthesis involve reduction of Au(III) followed by local covalent and effectively irreversible binding of Au atoms to albumin via Au-S bonds. For a mixed solution of BSA and more than 10-fold molar excess of HAuCl_4_ it is unlikely that at the end of the synthesis period there would be a significant portion of BSA molecules that do not bind at least one Au atom (i.e. BSA molecules with purely BSA-Alk characteristics). Nevertheless, we have carried out an electrophoretic control experiment (S1 Fig) confirming negligible presence of BSA-Alk-like specimen in BSA-AuNC samples. Fresh preparations of BSA-Alk and BSA-AuNC were strongly alkaline. Typically, pH of non-aggregated samples for spectroscopic measurements was re-adjusted with diluted HCl to 7 followed by dilution with deionized water, as specified in the following paragraphs. Eventually, samples prepared for far-UV CD (circular dichroism) and fluorescence measurement contained also co-dissolved NaCl at the concentration level of 1–2 mM. While spectrophotometric measurements allow one to verify BSA concentration (extinction coefficient for BSA at 280nm is approximately 43,824 M^-1^ cm^-1^) this approach would fail for both BSA-Alk and BSA-AuNC due to strong perturbations of UV absorption, especially in the latter case. Hence, careful pipetting of liquid protein samples of given concentration was used to prepare BSA-Alk and BSA-AuNC solution for further measurements. This approach, when assisted with the use of a precise balance, allows one to keep relative concentration uncertainty below 5%.

In order to trigger aggregation of albumin, the procedure described by Holm et al. was followed [31]. Namely, pH of freshly obtained BSA-AuNC sample was initially adjusted to 7.4 followed by addition of crystalline tris(hydroxymethyl)aminomethane (TRIS) to the final buffer concentration of 20 mM and re-adjustment of pH to 7.4 with diluted HCl. Samples were subjected to 96h-long (unless otherwise stated) quiescent incubation at 75 ^o^C resulting in the formation of aggregated BSA-AuNC (or ‘{BSA-AuNC}’: curly bracketed names are for aggregated albumin samples). {BSA} and {BSA-Alk} were obtained in a similar manner except that BSA-AuNC precursor was replaced with 23.8 mg/ml aqueous solution of freshly dissolved native BSA, or the BSA-Alk, respectively. For the control experiment requiring removal of traces of dissolved salts from {BSA-AuNC}, a 24h-long room temperature dialysis against 100-fold volume excess of deionized water using Spectra/Por Float-A-Lyzer G2 Dialysis Device with molecular weight cutoff of 3.5–5kD (Sigma-Aldrich) was applied.

### Spectroscopy

#### Fluorescence measurements

Luminescence of gold clusters in BSA-AuNC was excited at 365 nm in samples diluted 40-times with deionized water. For detection of amyloid-like fibrils with the Thioflavin T (ThT) fluorescence assay [33] stock samples of aggregates ({BSA}, {BSA-Alk}, {BSA-AuNC}) grown in 20 mM TRIS buffer, pH 7.4 (as described earlier) were diluted 33 times with deionized water without further pH adjustment; ThT was added to the concentration of 20 μM; ThT fluorescence was excited at 450 nm. All measurements were carried out using 10 mm quartz cuvettes on an AMINCO Bowman Series 2 luminescence spectrometer (bandpass 4 nm) at 25 ^o^C.

#### ATR-FTIR measurements

Liquid samples were transferred onto surface of single-reflection diamond ATR (attenuated total reflectance) accessory of Nicolet iS50 FTIR (Fourier transform infrared) spectrometer. Liquid suspensions were gently dried up in situ and infrared spectra of thus obtained films were collected. Typically, for a single spectrum 32 interferograms of 2 cm^-1^ resolution were co-added. Due to ambiguity in determining real values of refractive indexes of {BSA-Alk} and {BSA-AuNC} uncorrected ATR-FTIR data is shown. The Savitzky-Golay second derivative spectra (polynomial order 2, points of window 100) were calculated using Origin software. Routine processing of spectral data was carried out using GRAMS software (Thermo Scientific).

#### Circular dichroism measurements

For far-UV circular dichroism (CD) measurements of soluble (non-aggregated) samples, neutral pH stock solutions were diluted 100 times with deionized water (or GdnHCl solution, as specified in figure captions) to the final albumin concentration of 0.025 wt. % and subsequently placed in 1 mm quartz cuvettes. For near-UV CD measurements, the concentration of albumin was set at 0.75 wt. %. As aggregated protein tends to precipitate over time, it was desirable to carry out CD measurement of magnetically stirred samples of {BSA}, {BSA-AuNC}, and {BSA-Alk} which required 10 mm quartz cuvettes and further 10-times dilution of the sample to compensate for the increased optical density. All measurements were carried out at 25 ^o^C by accumulation and averaging of 10 CD spectra independently collected on Jasco J-815 S spectropolarimeter (Jasco, Japan).

#### Raman measurements

Raman spectra of commercial BSA and solid freeze-dried BSA-Alk (both in solid state) were collected on DXR Raman Microscope from Thermo Scientific equipped with a 780 nm laser operating at 10 mW power output. Due to very strong and persistent fluorescence, reasonable quality Raman spectra of BSA-AuNC samples could not be collected regardless of wavelength of the laser beam tested (including near-infrared laser–possibly due to two-photon-induced luminescence of BSA-AuNC [34]). For each Raman spectrum, 5 independent scans were averaged yielding final spectral resolution in the range between 4.7 and 8.7 cm^-1^. All spectra were corrected for residual background fluorescence using a 4^th^-order polynomial function.

### Susceptibility to trypsin digestion probed with sodium dodecyl sulfate-polyacrylamide gel electrophoresis (SDS-PAGE)

Fresh 1 mg/ml solution of trypsin (from Sigma-Aldrich) in 1 mM HCl (pH 3) was used. After establishing optimal conditions for the digestion assay, 50 or 1 μg/ml of trypsin was chosen for comparison of proteolysis patterns of samples. The proteolysis proceeded in 50 mM phosphate buffer, pH 7.4. The reaction was carried out in a volume of 100 μl on Eppendorf ThermoStat Plus at 37°C for 20 min. SDS-PAGE-based analysis of digested protein samples was carried out according to a method described by Laemmli [35]. Immediately after proteolysis, 50 μl of a sample was added to 50 μl of SDS-PAGE sample buffer (125 mM TrisHCl, pH 6.8, 20% glycerol, 4% SDS, 0.1% bromophenol blue and incubated at 100°C for 3 min. Subsequently, 7.5 μL portion of protein sample (corresponding to 7.5 μg of BSA per lane) was loaded on 12% polyacrylamide gel. The electrophoresis was carried out using Hoefer SE250 Mini-vertical gel electrophoresis unit and PS600 Power Supply. The gel was run in running buffer (25 mM TrisHCl, 192 mM glycine, 0.1% SDS, pH 8.3) at a constant current of 30 mA. Protein bands were visualized by Coomassie staining. Quantitative densitometric measurements of SDS-PAGE bands’ intensities were carried out using free ImageJ software from National Institutes of Health, Bethesda, Maryland, USA (https://imagej.nih.gov/ij/, [36]).

### Imaging

#### TEM

For TEM imaging, 400 mesh copper grids covered with Formvar and carbon (Ted Pella) were used. 10 μL portions of appropriately diluted samples (at 2.5, 1.25, or 0.25 BSA w/v %) were placed on grids for 40 s. Some of the samples (as indicated in figure captions) were negatively stained for 25 s with 1 wt. % uranyl acetate (SPI Supplies). Grids were dried at room temperature and examined using high performance transmission electron microscope JEM 1400 (JEOL Co.) equipped with 11-megapixel TEM camera MORADA G2 7(EMSIS GmbH).

#### SEM

Scanning electron microscopy (SEM) images were obtained on Zeiss Ultra Plus scanning field emission microscope at 2 mm working distance with 2 kV accelerating voltage. Samples were prepared as follows: a 5 μL portion of sample solution was placed on epi-ready surface of silicon wafer. After 30 s, the excess of liquid was removed with filter paper. Prior to imaging, deposited samples were coated with 10 nm thick carbon layer.

#### AFM

Collected samples for atomic force microscopy (AFM) imaging were diluted 60 times with deionized water. A small droplet (8 μl) of thus obtained solution was swiftly deposited onto freshly cleaved mica and left to dry overnight. AFM tapping-mode measurements were carried out using Nanoscope III atomic force microscope (Veeco, USA) and TAP300-Al sensors, resonance frequency 300 kHz (BudgetSensors, Bulgaria).

#### Optical microscopy

Each sample was placed on silicon wafer and left to dry under ambient conditions. For the acquisition of optical images of {BSA-AuNC} Nikon Eclipse LV100ND microscope was employed. The imaging was carried out in reflection mode using differential interference contrast (DIC). Distances in the image plane were calibrated by imaging a 10-μm scale bar standard.

## Results and discussion

### Conformational state and stability of AuNC-binding albumin

**Fig 1:**
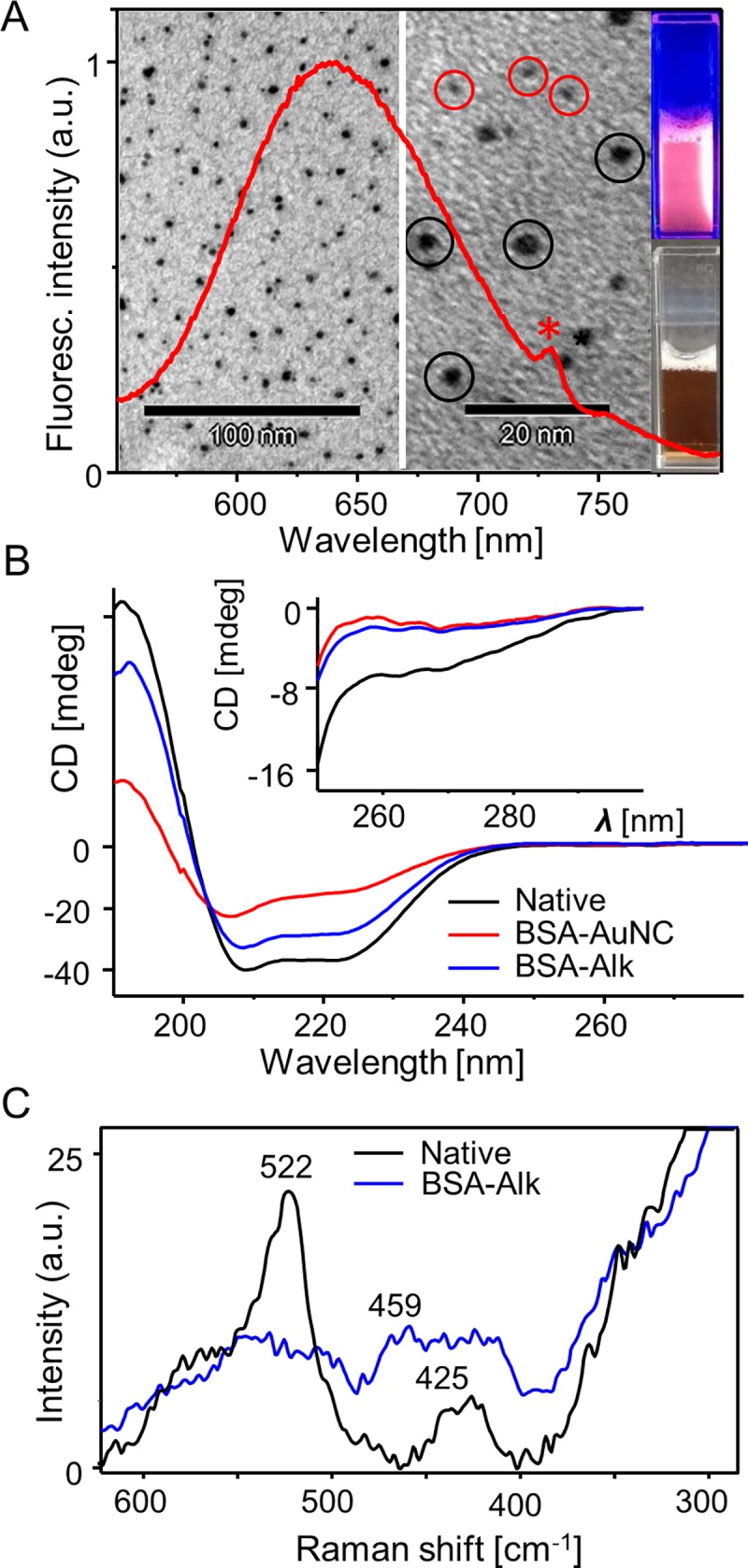
(A) Luminescence emission spectrum of BSA-AuNC (λ_exc._ = 365 nm) overlaid on unstained TEM images of BSA-AuNC; AuNCs of diameter below 1 nm are marked with red circles whereas larger non-fluorescent AuNPs are in black circles. Photographs of BSA-AuNC liquid sample illuminated with 365 nm UV light (top), and in daylight (bottom) are on the right. (B) Far-UV CD spectra of native BSA, BSA-Alk, and BSA-AuNC collected at the same protein concentration and pH 7; inset shows the corresponding near-UV CD spectra. (C) Raman spectra of native BSA and BSA-Alk (laser line 780 nm).

BSA-AuNC samples prepared according to the protocol developed by Xie and colleagues [5] exhibited the characteristic red luminescence when excited at 365 nm (Fig 1, panel A). The corresponding TEM images of unstained conjugate reveal the presence of tiny AuNCs along with larger objects of the size of several nanometers corresponding to non-luminescent gold nanoparticles (AuNPs) which form as a byproduct. For subsequent conformational studies ‘BSA-Alk’—albumin subjected to a prolonged incubation in alkaline environment but in the absence of HAuCl_4_ was prepared as an additional control sample. Far-UV CD spectra of BSA-AuNC, BSA-Alk, and unmodified, native BSA collected under identical conditions are shown in Fig 1, panel B. As is expected for a stably folded α-helical protein, the spectrum of native BSA features two minima of approximately similar intensity at ca. 208 and 222 nm. The roughly 20% decrease in ellipticity observed for BSA-Alk reflects partial unfolding of the helical structure. This indicates that alkaline-induced denaturation of BSA is irreversible since pH of all samples was pre-adjusted to neutral prior to CD measurements. We note an even more pronounced (by approximately 50%) decrease in helicity in BSA-AuNC. This is accompanied by shifting below 208 nm of the ππ* transition component, and the nπ* transition band at 222 nm becoming disproportionately attenuated. The corresponding near-UV CD spectra reporting on stability of tertiary contacts between aromatic side-chains are shown in the inset of Fig 1, panel B. The decay of ellipticity around 270 nm observed for BSA-Alk and BSA-AuNC is more dramatic than the changes observed in the far-UV region. Although it is tempting to interpret these observations in terms of a molten globule-like state of BSA-Alk and BSA-AuNC (fluctuating partly disordered molecule with residual secondary structure [37]), other factors could contribute to the flattening of near-UV CD spectra, as well (for example, tyrosine dimerization, breakage of CD-active disulfide bonds [38]). Evidence of significant disruption in disulfide bonding occurring upon exposure of BSA to the alkaline environment was provided by Raman spectroscopy. There are 17 disulfide bonds per folded BSA monomer [16]. Raman-active stretching *v*(S-S) vibrations [39] around 522 cm^-1^ are prominent in the corresponding Raman spectrum of native albumin (Fig 1, panel C) but almost disappear from the spectrum of BSA-Alk (measurement of BSA-AuNC spectrum proved unfeasible due to strong and persistent fluorescence–see the Materials and Methods).

**Fig 2:**
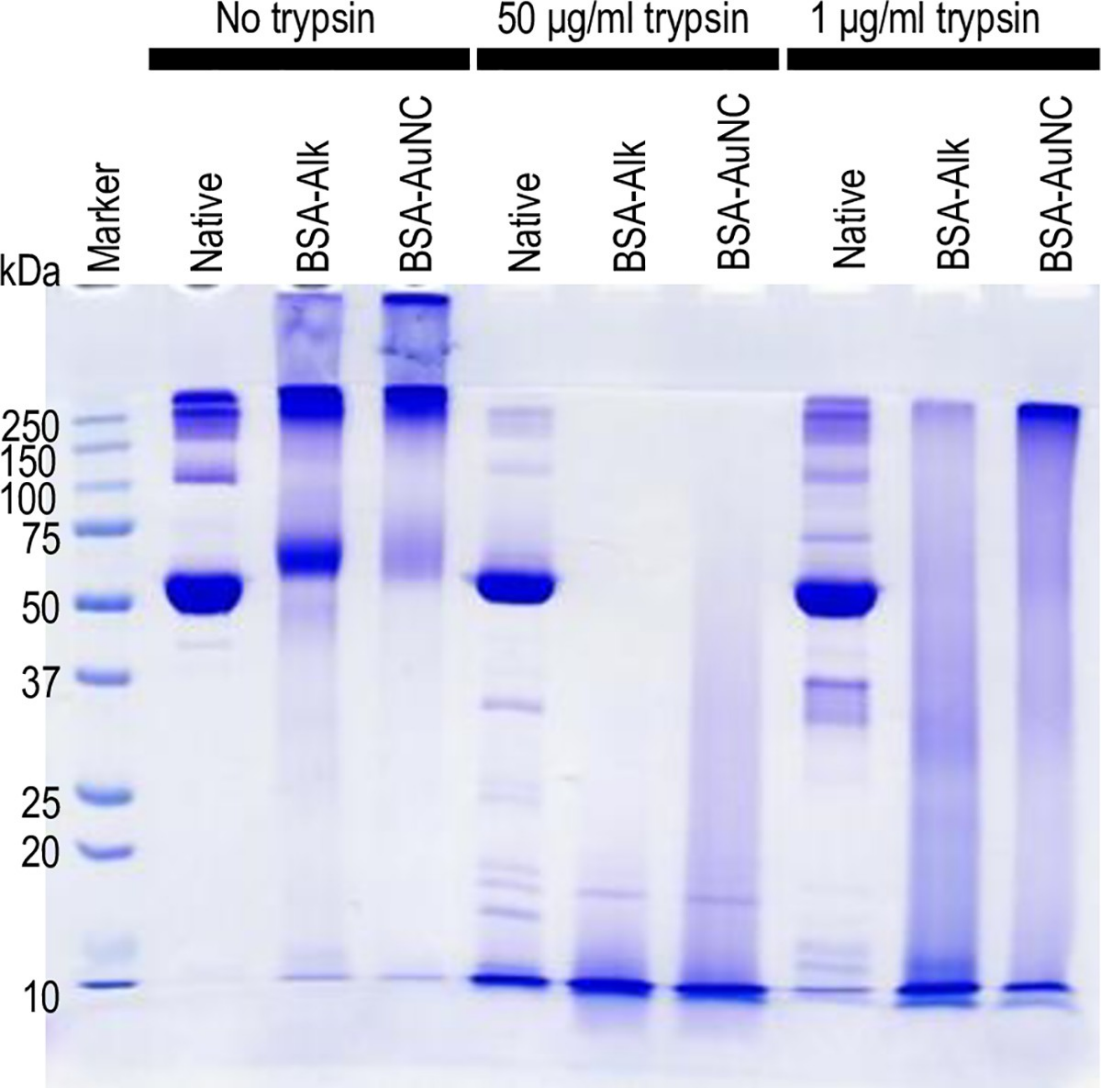
SDS-PAGE analysis of susceptibility to digestion by trypsin of BSA, BSA-Alk, and BSA-AuNC under non-reducing conditions. A portion of each sample corresponding to 7.5 μg of BSA mass was loaded onto each lane.

These very initial results indicate that formation of BSA-AuNC conjugate is accompanied a significant loss of BSA’s native α-helical structure, loosening of tertiary contacts between aromatic residues, and an extensive breakage of disulfide bonds. Unsurprisingly, such drastic changes within albumin molecules occurring simultaneously on all the three structural levels are irreversible. The spectral Raman and CD characteristics of BSA-Alk provides unequivocal evidence that the alkaline environment of the synthetic protocol, rather than binding of AuNCs, is the primary factor responsible for the observed structural disruption of albumin. In such case, the high vulnerability of BSA-AuNC to trypsin digestion should also be observed for BSA-Alk. We have employed SDS-PAGE technique to verify this possibility. Results of limited proteolysis with trypsin of native BSA, BSA-Alk, and BSA-AuNC under non-reducing conditions are shown in Fig 2. Such conditions are expected not to interfere with local Au-S bonds responsible for docking AuNC within the albumin envelope [40]. In the absence of trypsin, all samples exhibit intensive bands above 250 kDa originating from largest oligomers incapable of entering the resolving gel. Intensity of the 66 kDa band assigned to BSA monomers decreases in the order BSA > BSA-Alk > BSA-AuNC for which it becomes particularly low. Only for BSA there is a weak but well resolved band at approx. 130 kDa (assigned to dimer). Digestion with trypsin results in a complete fragmentation of monomers and oligomers of BSA-Alk and BSA-AuNC whereas monomers of unmodified albumin remain resistant to proteolysis to a significant degree (unlike its oligomers as the lessening intensity of the band above 250 kDa suggests). The faint bands between 25 and 50 kDa corresponding to intermediate products of BSA proteolysis are absent in BSA-Alk and BSA-AuNC lanes (trypsin with its molecular weight of 23.3 kDa may be detectable only in high enzyme concentration lanes).

One characteristic distinction between BSA-Alk and BSA-AuNC is the ‘monomer’ band in non-digested samples which is much weaker for BSA-AuNC. In principle, an electrophoretic (or spectral) band selectively observed for BSA-Alk (and not for BSA-AuNC) could be employed to estimate the amount of BSA-Alk present in BSA-AuNC. However, using for this purpose the ‘monomer’ bands visible in BSA-Alk / BSA-AuNC lanes not treated with trypsin is problematic given the unspecific amounts of large oligomers unable to enter the resolving gel (Fig 2). This problem could be circumvented with a preliminary careful digestion at low trypsin concentrations. We have tested this approach and the results are reported in S1 Fig. We found that after gentle digestion facilitating protein entry into the resolving gel the ‘monomer’ bands disappear but another double band at approx. 15 kDa is observed selectively in BSA-Alk lanes. According to densitometric measurements the concentration of protein species with BSA-Alk-like trypsin-digestion characteristics does not exceed 9% of BSA-AuNC sample (S1 Fig).

**Fig 3:**
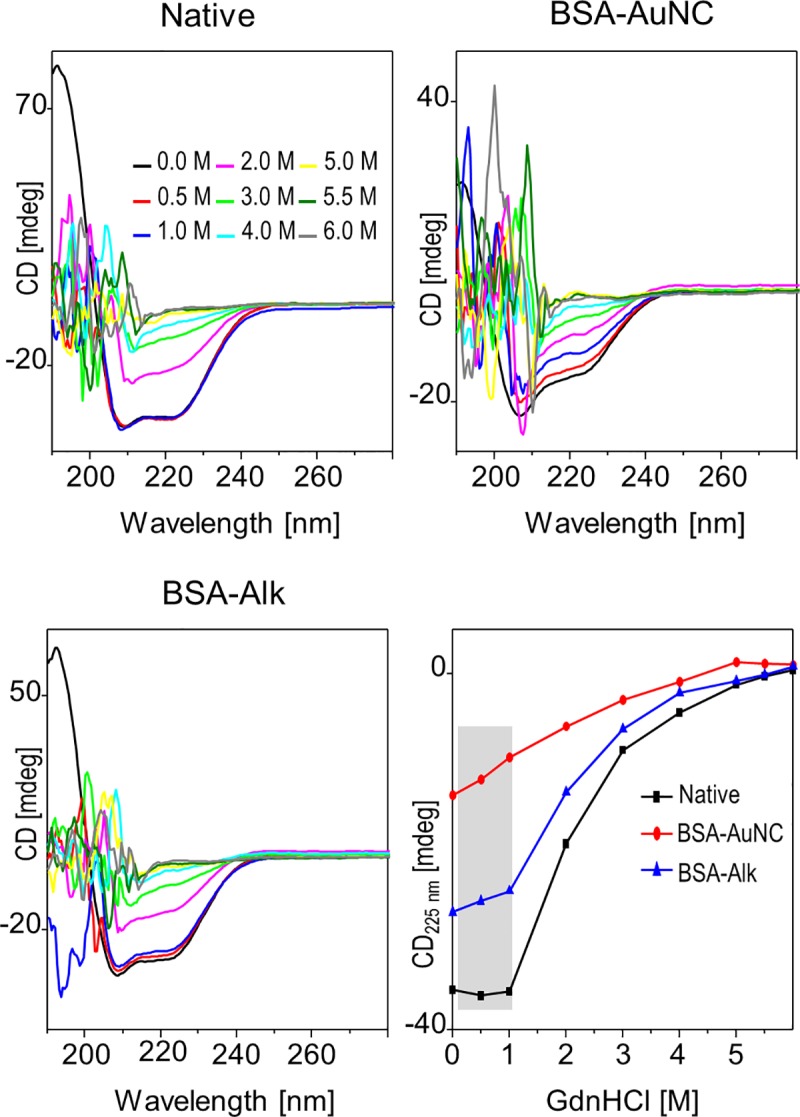
Conformational stability of BSA-AuNC complex. Titration of BSA-AuNC, native BSA, and BSA-Alk with GdnHCl at pH 7 and 25 ^o^C monitored with far-UV CD. In the bottom right panel, quantitative plots correspond to GdnHCl-dependences of CD signals at 225 nm. Plasticity of secondary structures decreases in the order: BSA-AuNC > BSA-Alk > native BSA. The corresponding low GdnHCl concentration range is marked with the shadowed area.

Coupling stepwise chemical denaturation to a spectroscopic structural probe often serves as an insightful approach to protein stability. Chib et al. monitored GdnHCl-induced unfolding of BSA-AuNC by analyzing fluorescence of tryptophan residues [21]. Interpretation of the data in terms of local unfolding-related changes in polarity around these residues may be challenging due to complex and poorly understood resonance energy transfers involving AuNCs [41]. In our approach, far-UV CD spectroscopy was used instead as a straightforward gauge of protein secondary structure. Fig 3 presents CD spectra of BSA, BSA-AuNC, and BSA-Alk collected in the presence of increasing concentration of GdnHCl. In the absence of denaturant, all three samples contain various amounts of residual α-helical structure (Fig 1, panel B). Low concentrations of GdnHCl (up to 1 M) virtually do not affect spectra of unmodified BSA which contrasts with the response of BSA-AuNC and BSA-Alk already undergoing denaturation. In all three cases, the spectrum closest to half of the initial CD intensity is obtained in the presence of 2 M GdnHCl, while the continuous decrease in ellipticity levels off only above 4 M concentration of denaturant, the corresponding CD spectra become completely flat in the spectrally accessible range (i.e. not eclipsed by strong deep UV absorption by GdnHCl) indicating that albumin has acquired the random coil conformation. These tendencies are reflected in the qualitative plots of CD signal dependence on denaturant concentration shown also in Fig 3. The vulnerability of BSA-AuNC to low denaturant concentrations is likely to be another consequence of the structural plasticity and marginal stability that underlie the susceptibility to trypsin. A similar course of the CD signal dependence on GdnHCl concentration is observed for BSA-Alk. The slow almost linear decrease in absolute ellipticity values of BSA-AuNC titrated with GdnHCl contrasts with the sharper and cooperative denaturation of unmodified BSA and could be interpreted as a manifestation of conformational heterogeneity.

**Fig 4:**
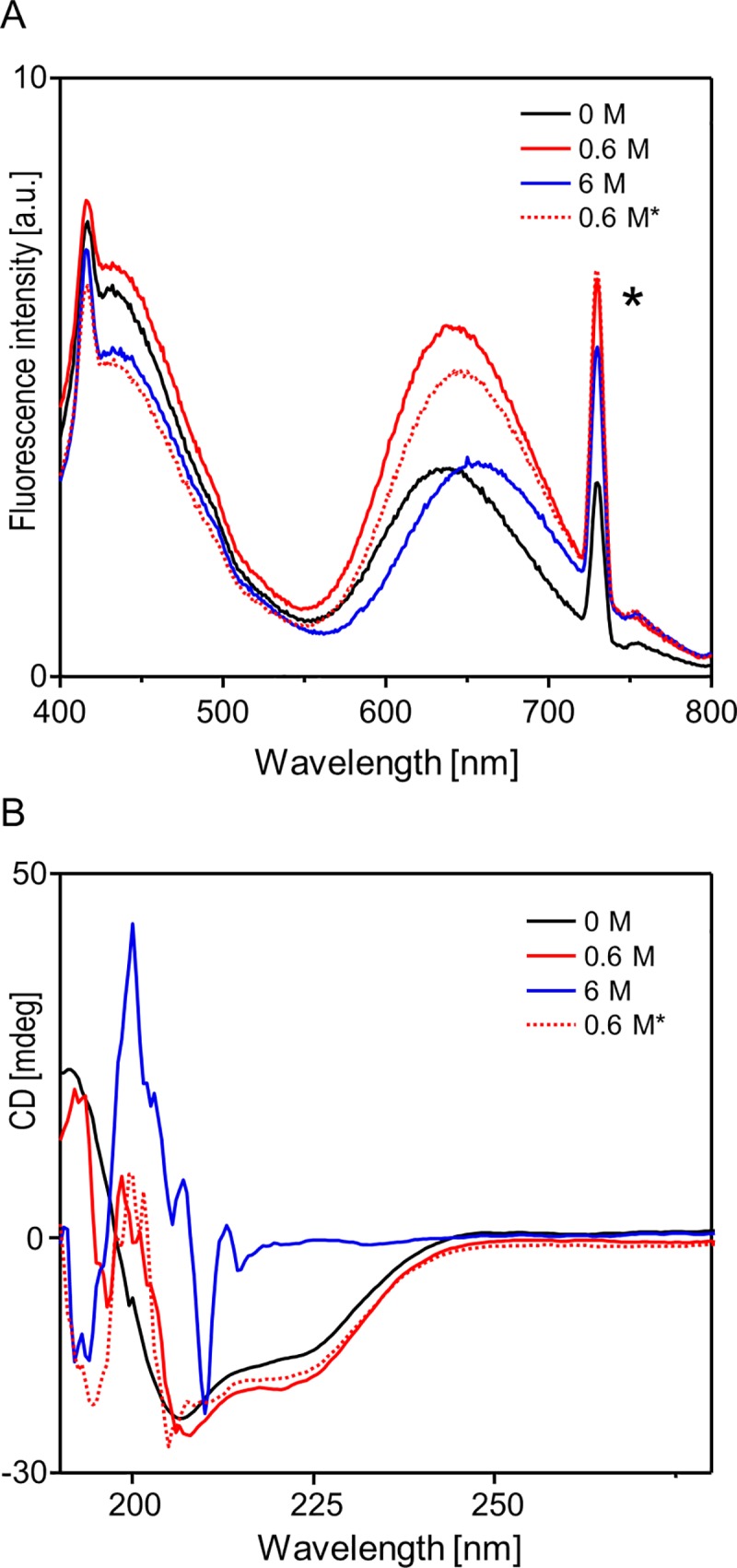
Reversibility of GdnHCl-induced unfolding of BSA-AuNC probed by luminescence of AuNC excited at 365 nm (A) and far-UV CD (B). The 0.6 M* label corresponds to initially 10-times more concentrated BSA-AuNC samples subjected to 6 M GdnHCl and subsequently ‘refolded’ by 10-times dilution with water; sharp peak marked with an asterisk corresponds to scattered 2*λ_exc_ light. The region of CD spectra collected in the presence of 6M GdnHCl corresponding to shortest wavelengths is perturbed due to strong UV absorption by the denaturant.

The guanidinium-induced denaturation of BSA-AuNC is profound but fully reversible both in terms of secondary structure of albumin and luminescent properties of AuNCs. In Fig 4, emission (excited at 365 nm) and far-UV CD spectra of BSA-AuNC dissolved in water, diluted 0.6 M GdnHCl (before full unfolding), 6M GdnHCl (at full unfolding), and back in 0.6 M GdnHCl (i.e. after 10-times dilution of the fully denatured sample) are juxtaposed. The dilution-induced refolding is complete. It should be stressed that the course of GdnHCl-induced changes in luminescence of BSA-bound AuNCs presented in this work is only in a partial agreement with the data published earlier ([21]). In accordance with the study by Chib et al., the emission band at 640 nm undergoes a monotonic redshift with the increasing GdnHCl concentration. In this work, however, we have also detected a transient and previously unreported increase in emission intensity (see S2 Fig). The luminescence intensity levels of BSA-AuNC in water and in 6 M GdnHCl are similar. Presently, we can only speculate that an increase in fluctuations induced by low denaturant concentrations could damp interactions within BSA-AuNC responsible for intrinsic quenching of luminescence. The full reversibility of BSA-AuNC red luminescence is a strong evidence that AuNCs are anchored within the albumin envelope by strong guanidinium-resistant covalent bonds, as postulated earlier [40]. Otherwise, should nanoclusters be shed by BSA during unfolding, these metastable entities would immediately and irreversibly cluster into larger non-luminescent particles, as in the case of trypsin-induced degradation of BSA-AuNC [18].

In the state bound to luminescent AuNCs, BSA is a significantly disordered molecule retaining only a fraction of its native α-helical conformation. With most of BSA’s disulfide bonds hydrolyzed by the alkaline environment of conjugate’s synthesis (Fig 1, panel C), a newly formed AuNC could possibly be bound to multiple thiol groups from broken–SS–bridges [42–43] and play an analogous conformation-restricting role. As partly disordered protein molecules are thought to be prone to aggregation and formation of amyloid-like fibrils [27], in the following part of this study, we have focused on self-association behavior of BSA-AuNC.

### Aggregation of BSA-AuNC

**Fig 5:**
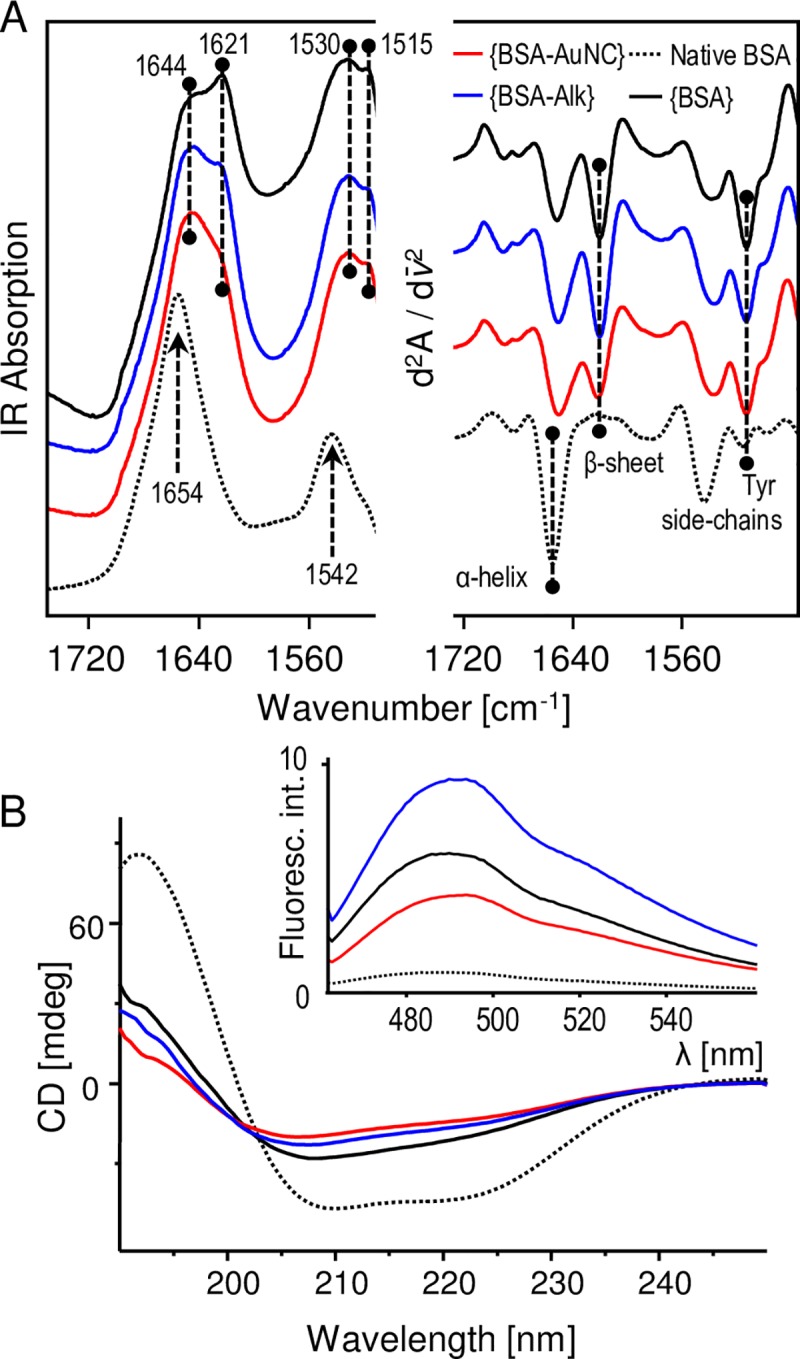
(A) ATR-FTIR spectra (left: original absorption, right: corresponding second derivative) of aggregates: {BSA-AuNC}, {BSA-Alk}, and {BSA} obtained through 96h-long incubation of corresponding soluble precursors at pH 7.4, 75 ^o^C compared with the spectra of native BSA; (B) The corresponding far-UV CD spectra and fluorescence emission spectra after staining with Thioflavin T (inset), the assignment of colors is the same as in panel (A).

Luminescent BSA-AuNC conjugate along with BSA-Alk and unmodified native BSA (as controls) were subjected to prolonged incubation in TRIS buffer: the condition known to favor spontaneous self-association of unmodified BSA molecules into amyloid-like fibrils [31]. Infrared spectroscopy was used for an initial assessment of the changes in the secondary-structure of albumin after 96 hours (Fig 5, panel A). Compared with the spectrum of native BSA, the main spectral component of the amide I vibrational band of aggregated conjugate ({BSA-AuNC}) is broadened and red-shifted (from 1654 cm^-1^ to approx. 1644 cm^-1^) implying a degree of denaturation of the native structure. Moreover, the low-frequency shoulder at ca. 1621 cm^-1^ (clearly visible in the corresponding second derivative spectra) indicates the presence of non-native β-sheets. The lack of the high frequency component above 1680 cm^-1^ suggests that these conformations are distinct from intermolecular antiparallel β-sheets often found in amorphous aggregates [44]. Comparison with the spectra of other control aggregate samples: {BSA} and {BSA-Alk} reveal even higher contents of β-sheet structure. Therefore the degree of covalent perturbation of ‘substrate’ albumin does not correlate with its tendency to form non-native β-sheets. In the following step, far-UV CD was employed to examine the samples (Fig 5, panel B). In unison with the infrared data, the CD spectra point to decreased content of native helical structure in all types of aggregates (reflected by the flattened CD curves), although the putative presence of β-conformation is less obvious, possibly due to scattering of UV-light on larger aggregates. ThT is an amyloid-fibrils-specific molecular rotor with the quantum yield of fluorescence increasing sharply upon docking on amyloid surfaces [33, 45]. We have used ThT fluorescence to probe presence of amyloid fibrils in aggregates. Inset in the panel B of Fig 5 shows the emission spectra of ThT-stained aggregates (along with native BSA as a control) excited at 450 nm. While all aggregates enhance ThT emission in comparison to native BSA, the degree of this enhancement (by approx. an order of magnitude) is relatively low; typically a complete transition of globular protein into amyloid fibrils is expected, under optimal conditions, to enhance ThT emission by 2–3 orders of magnitude [45]. We note that out of all the three types of aggregates, intensity of ThT fluorescence in the presence of {BSA-AuNC} is lowest.

**Fig 6:**
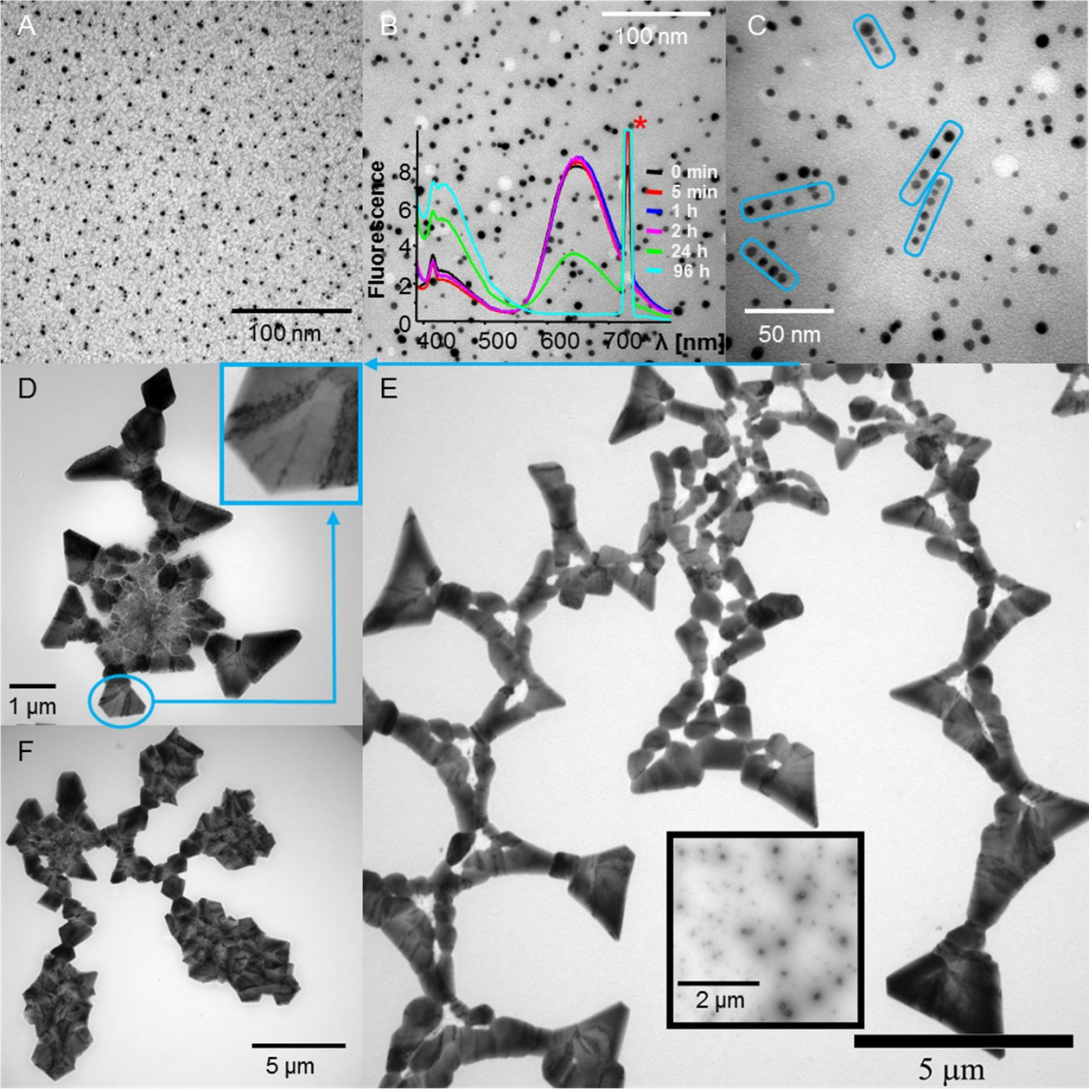
TEM images of BSA-AuNC complexes before (A), and after aggregation (B-F). The singly-dispersed AuNCs (A) associate into elongated entities upon prolonged incubation of BSA-AuNC complexes at pH 7.4, 75 ^o^C–e.g. the encircled groups in panel (C). The progressing aggregation is accompanied by a gradual decrease in fluorescence intensity (excited at 365 nm), as reflected by the emission spectra overlaid in panel (B), the intense spike marked with (*) corresponds to scattered excitation beam. {BSA-AuNC} form regular superstructures visible on larger length scales (D-F). In the magnified image shown in inset of panel (D), the dark streaks are revealed to consist of elongated groups of AuNCs. The self-association behavior of {BSA-AuNC} is not observed for aggregates of BSA alone (inset image in panel (E) obtained after negative staining with uranyl acetate).

The picture of the structural state of {BSA-AuNC} emerging from the spectroscopic characterization is therefore complex. The content of disordered / β-sheet conformations increases at the expense of the native structure, and this appears to coincide with the formation of aggregates capable of enhancing ThT emission in the manner similar to amyloid fibrils. As non-aggregated BSA-AuNC is vulnerable to trypsin digestion whereas amyloid fibrils are generally resistant to proteases [46], we used again controlled proteolysis with trypsin and SDS-PAGE to check how aggregation affects this property of BSA-AuNC. According to the data placed in S3 Fig, treatment with concentrated trypsin of {BSA-AuNC}, as well as {BSA} and {BSA-Alk} under non-reducing conditions results in only partial digestion of largest aggregates. We arrived at some of the key findings of this work while examining morphologies of {BSA-AuNC} using different microscopic techniques. TEM images of BSA-AuNC conjugates before and after aggregation are shown in Fig 6, panels (A), and (B-F), respectively. The chaotically spread singly-dispersed small AuNCs and larger non-luminescent AuNPs are the typical TEM features of non-aggregated BSA-AuNC. The aggregation process has two dramatic consequences, namely: [i] gradual but significant decrease in luminescence of AuNCs (see the emission spectra superimposed on panel B) occurring on the timescales corresponding to the self-association of protein molecules, [ii] ordering of gold clusters and particles into elongated entities shown in Fig 6, panel C. A similar effect has been reported earlier for amyloid aggregates decorated with AuNPs [47]. The small size of luminescent AuNCs makes their images resolve poorly compared to larger AuNPs hampering efforts to quantify them with TEM. Tiny AuNCs are still visible in TEM images of {BSA-AuNC} indicating that recrystallization of bright AuNCs into dark AuNPs is unlikely to be the sole reason of the decrease in red luminescence. Hypothetically, even subtle changes in binding interactions between AuNCs and BSA following the conformational transition in protein backbone could have a similar effect [48]. Only when imaging of {BSA-AuNC} structures was conducted at much lower magnification we were able to observe the striking morphological features of these aggregates. Panels (D-F) in Fig 6 present giant superstructural arrangements of {BSA-AuNC} accessing geometries absent from the palette of known (to our best knowledge) proteinaceous assemblies. The triangular edges are visible in both fractal-like assemblies reminiscent of leaves (see panels (D) and (F)) and in twisted chain-like superstructures depicted in panel (E). In each case, the whole content of {BSA-AuNC} appears to participate in these motives. Consequently, all AuNCs and AuNPs were entrapped, enabling imaging of these aggregates without the need for negative staining. Elongated assemblies of AuNCs and AuNPs retain the streak-like appearance as shown in inset in Fig 6, panel D. Also, it is clear from the control images of negatively-stained aggregates of BSA (inset in Fig 6, panel E) that the amyloidogenic self-assembly of unmodified protein (studied thoroughly in earlier works–e.g. [31]) does not produce similar forms.

**Fig 7:**
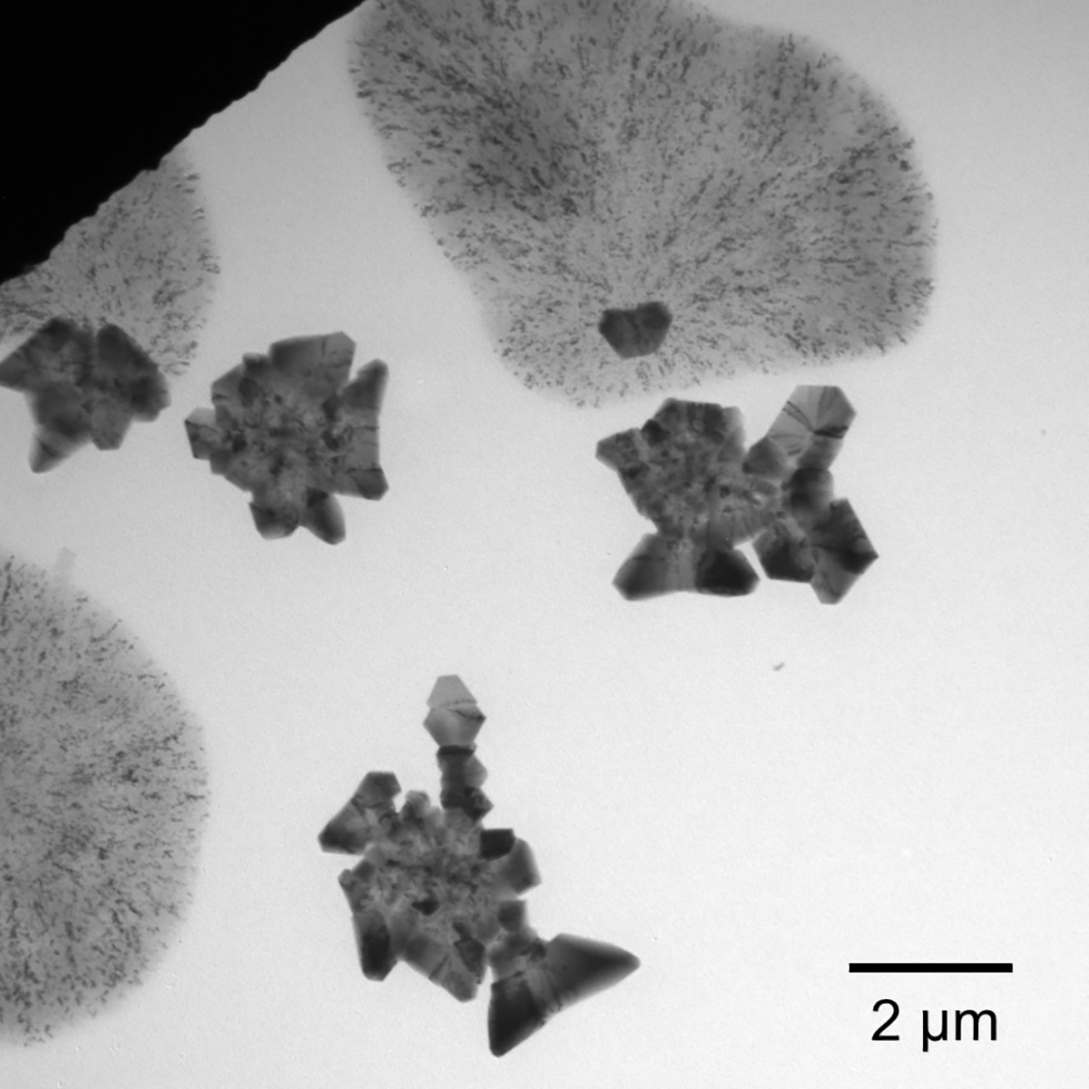
Specimen of early diffuse {BSA-AuNC} aggregates co-existing with mature superstructures, as observed by TEM. The mold-like entities reveal AuNCs being already pre-associated into stretched elongated groups before the superstructural self-assembly is completed.

An extensive survey of the morphological types of the {BSA-AuNC} confirmed that the chain-like and fractal-like assemblies of triangular building blocks connected with twisted tape-like forms are omnipresent. However, we were also able to observe occasionally different types of “diffuse” aggregates (as shown in Fig 7) co-existing with superstructures. Within these mold-like entities, Au intrusions (either as AuNCs or AuNPs) are associated into stretched elongated groups which, at least on the small local scales, are parallel to each other. At this point, we may only speculate that the diffuse forms constitute early pre-assembled {BSA-AuNC}, i.e. image in Fig 7 is likely to constitute a snapshot of an incomplete interrupted transition. We have employed other microscopic techniques to probe {BSA-AuNC} morphology, as shown in S4 Fig. In order to verify whether traces of buffer and salts co-crystalizing on the substrates used for TEM could contribute to the unusual appearance of {BSA-AuNC}, additional control experiments were carried out. Mature samples of {BSA-AuNC} were subjected to prolonged dialysis against larger volumes of deionized water prior to drying and morphological analysis. The corresponding images are shown in S5 Fig. We note the persistence of all the key morphological traits mentioned earlier: triangular building blocks, twisted connecting chains, and fractal patterns implying that these giant superstructures are, indeed, built of metal-intrusion-doped protein aggregates.

**Fig 8:**
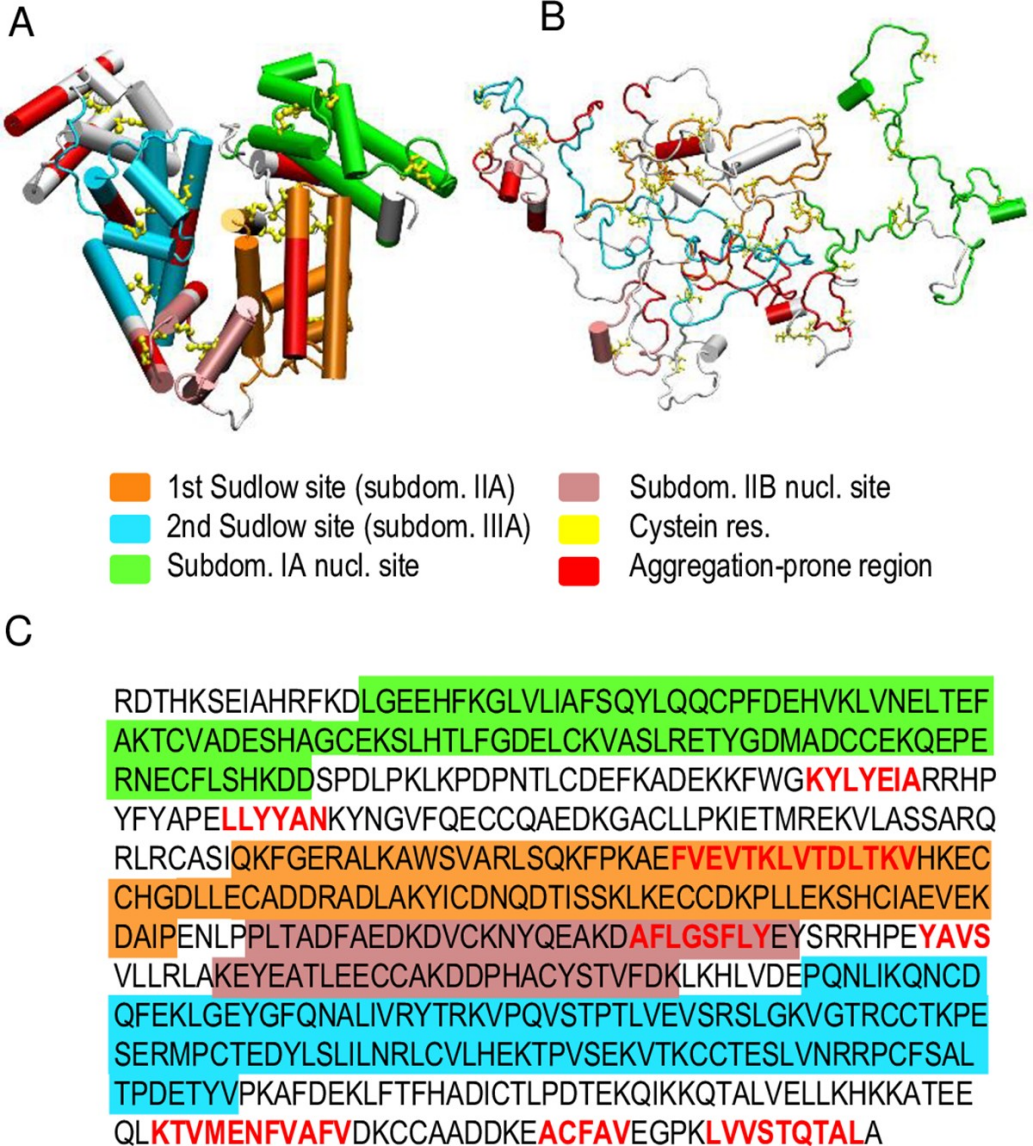
BSA: a 3D structure of native protein (A), PDB entry 3V03 [51], and an MD-generated snapshot of alkaline-disordered state (B). Various colors mark regions involved in AuNC-binding according to [22]. Regions with high propensity to aggregate mapped according to Tango algorithm [49–50]. Full amino acid sequence of BSA with likewise highlighted AuNC-binding and aggregation-prone regions (C). For MD-derived snapshot CHARMM package [52] and implicit solvent FACTS22 method [53] were used. Disordered conformations were obtained by 10-ns-long thermal unfolding of BSA with all disulfide bonds reduced (550 K, pH 12). Visualization was done with VMD program [54].

The formation of BSA-AuNC conjugates takes place under conditions which not only destabilize the native state of serum albumin but also trigger irreversible changes in its chemical composition. The alkaline environment of this process alone is sufficient to cause vanishing of tertiary contacts and to reduce the native helical content in BSA, as was demonstrated using CD spectroscopy. It is evident from the remarkable intensity decrease of Raman band corresponding to *v*(S-S) stretches that most of disulfide bonds are hydrolyzed upon the alkaline treatment. This, in turn, causes disarray of higher order structures rendering albumin molecules partly disordered and vulnerable to proteolysis with trypsin. In light of these findings, certain computational and experimental approaches put forward on the assumption that BSA within the complex with AuNC remains in a quasi-native state become problematic (Fig 8). For example, in a disordered state of base-denatured albumin specific thiol groups and surviving disulfide bonds at which gold clusters may dock are likely to be different from those most accessible on the surface of the native state [22]. The partly disordered AuNC-bound state of albumin does, however, have some very interesting traits. The complete chemical denaturation with guanidinium is fully reversible: the AuNCs are not ‘lost’ during unfolding / refolding stages but may, in fact, enhance efficiency of renaturation by restricting fluctuations of cluster-bound chains. Despite having most of the–SS–bonds gone, BSA-AuNC is less prone to form non-native β-sheets than unmodified BSA (which itself is a marginally amyloidogenic protein with ca. only 15% of the overall amino acid sequence classified as predisposed to aggregation according to the Tango algorithm (Fig 8) [49–50]). Nevertheless, BSA-AuNC do self-associate even though the process is distinct from the well-known transition pathways leading to amyloid-like or amorphous aggregates. This is reflected by the absence of canonical amyloid-like fibrils in TEM images, marginal β-sheet content according to CD and infrared spectroscopy, and low fluorescence enhancement in ThT.

The here demonstrated new type of giant supramolecular protein assemblies of {BSA-AuNC} reveals a range of fascinating morphologies quite different from known superstructures of amyloid fibrils [55–58]. The transition is accompanied by a decrease in luminescence of trapped AuNCs and the mechanism of this process, as well as of the morphological transition itself are not understood at this point. The puzzling observation that aggregation in the presence of diluted TRIS buffer and at the close-to-neutral pH leads to decrease of AuNC luminescence, whereas the complete denaturation of BSA-AuNC in 6 M GdnHCl does not (Figs 4A and 6B) draws attention to the linear groupings of AuNCs/AuNPs visible in TEM images of {BSA-AuNC}. Hypothetically, a self-quenching mechanism facilitated by proximity of metallic particles in aggregated protein could be involved. Our results suggest that stabilization of the singly-dispersed state of BSA-AuNC, rather than of the remnants of albumin native structure, is more important for maintaining conjugate’s luminescent properties. Further studies are needed to provide a satisfactory understanding of the unusual self-association behavior of {BSA-AuNC} and relationship between the structural state of albumin and the photophysics of AuNCs it is enveloping. Additional studies on the conformational state of albumin binding AuNCs will facilitate various applications of these systems and help tune their optical properties.

## Conclusions

Albumin molecules conjugated to luminescent AuNCs are trapped within a fragile and partly disordered state with a marginal tendency to form amyloid-like fibrils, but a strong propensity to self-assemble into giant superstructures with surprisingly low β-sheet content. Protein-mediated self-association of AuNCs appears to affect the emission intensity to a much higher degree than the structural transitions within singly-dispersed AuNC-bound albumin. The characteristic properties of AuNC-bound BSA such as the conformational disorder and susceptibility to proteases are likely to arise from the disarray of protein’s covalent structure caused by the basic environment of synthesis of BSA-AuNC, rather than by binding to AuNCs. In particular, this holds true for the extensive hydrolysis of native disulfide bonds. The extend of the actual structural disruption within AuNC-enveloping BSA molecules should be taken into account in future studies aimed at elucidating binding mechanisms between metal and protein.

## Supporting information

S1 Fig Estimation of the upper limit of residual albumin with BSA-Alk-like characteristics in BSA-AuNC samples: SDS-PAGE of partially digested BSA-Alk and BSA-AuNC. SDS-PAGE analysis of BSA-Alk (marked with blue arrows, lines 2, 4, 6, 8) and BSA-AuNC (marked with red arrows, lines 3, 5, 7, 9) gently digested with diluted trypsin (0.1 μg/ml) at 37 ^o^C for various periods of time (specified over the lanes). Each lane was loaded with a 6,75 μg portion of digested albumin; other details as specified in the main article. The gentle digestion procedure facilitated fragmentation of large aggregates which otherwise would not enter the gel while conserving distinct patterns characteristic for BSA-Alk and BSA-AuNC. Specifically: a clear distinction between samples of BSA-Alk and BSA-AuNC digested for 5 minutes (lanes 2, and 3, respectively) is observed. The double band around 15 kDa is resolved only in trypsin-treated BSA-Alk samples. Quantitative densitometric measurements of the band’s intensity for BSA-Alk and BSA-AuNC (analyzed areas are marked with yellow windows) were carried out using ***ImageJ*** software. The obtained intensity ratio of the BSA-Alk and BSA-AuNC areas was approximately 10:1. This implies that the concentration of protein species with *BSA-Alk-like trypsin-digestion characteristics does not exceed 9%*. Importantly, the “BSA-Alk-like trypsin-digestion characteristics” **does not equal being BSA-Alk**. Although similar situation is observed for protein samples digested for 10 minutes, we note that with the digestion time increasing further, the contrast decreases due to fragmentation of larger BSA-AuNC species.
(PDF)

S2 Fig Titration of BSA-AuNC with GdnHCl: luminescence of AuNCs. Effect of increasing concentration of GdnHCl on luminescence of BSA-AuNC excited at 365 nm (spectra collected at pH 7 and 25 ^o^C); the cut-off peak marked with an asterisk corresponds to scattered 2*λ_exc_ light. Quantitative dependences of luminescence intensity at 640 nm and luminescence λ_max_ on GdnHCl concentration are shown in the inset.
(PDF)

S3 Fig SDS-PAGE analysis of susceptibility to digestion by trypsin of aggregates: {BSA}, {BSA-Alk}, and {BSA-AuNC} under non-reducing conditions. Experimental conditions were the same as used for SDS-PAGE analysis described in Fig 2 of the main article. **Comment:** Aggregate samples consisted mostly of high-molecular-weight species insoluble in SDS-PAGE buffer. However, these aggregates turned out to be sensitive to a degree to trypsin digestion. The higher concentration of trypsin used in the assay led to degradation and simultaneous release of smaller fragments of diverse sizes that revealed themselves as smears along the gel’s lanes. On the other hand significant portions of oligomers remained at the resolving gel boundary for each type of sample. The lower concentration of trypsin was ineffective in fragmentation of largest aggregates.
(PDF)

S4 Fig Morphology of {BSA-AuNC}. Aggregates were observed using optical microscopy (A) and SEM (B-D). The fractal-like arrangements of aggregates were also observed using tapping mode AFM on mica support as shown in (E). Cross-sections of selected specimen of aggregates obtained from the corresponding AFM height images are displayed in panel (F). **Comment:** The large size of aggregates enabled detecting them even using optical microscopy: panel (A). The higher resolution afforded by SEM confirmed the presence of triangular forms (B-C) also in the case of aggregates deposited on silicon wafer (instead of Formvar/carbon substrates used for TEM imaging). The fractal arrangements of {BSA-AuNC} were also observed in SEM and AFM images vide amplitude AFM image in panel (E) collected on a mica substrate, although the latter technique is best-suited for flat aggregates that do not vary greatly in depth, as {BSA-AuNC} tend to do. When the acquisition of proper AFM images was possible, cross-sections were obtained from the corresponding height images (F). Such sufficiently flat specimen were estimated to be on average 10–25 nm thick, i.e. remarkably more than the diameter of typical amyloid fibrils.
(PDF)

S5 Fig Control data: morphology of {BSA-AuNC} after removal of salts through dialysis, morphology of {BSA-Alk}. Control TEM images of {BSA-AuNC} subjected to preliminary 24h-long dialysis against 100-fold excess of deionized water (A-B). The key morphological features of the aggregates including twisted superstructures and diffuse early aggregates are clearly preserved when traces of salts are removed. (C) TEM image of {BSA-Alk}. **Comment:** Interestingly, higher abundance of the diffuse forms in dialyzed aggregates suggests that the kinetics of maturation of {BSA-AuNC} may strongly depend on minute variations in ionic strength. The presence of clumped entities in {BSA-Alk} (C) suggests that the perturbation in BSA caused by the alkaline treatment may be a key factor predisposing the protein envelope to self-associate which clearly plays a significant role in the self-assembly pathway of {BSA-AuNC}.
(PDF)
