# Supplementary material for: Revisiting the conformational state of albumin conjugated to gold nanoclusters: A self-assembly pathway to giant superstructures unraveled
Source: PLoS One. 2019 Jun 27;14(6):e0218975. doi: 10.1371/journal.pone.0218975 (PMC6597083; doi:10.1371/journal.pone.0218975)
Supplement: S1 Fig — SDS-PAGE analysis of BSA-Alk (marked with blue arrows, lines 2, 4, 6, 8) and BSA-AuNC (marked with red arrows, lines 3, 5, 7, 9) gently digested with diluted trypsin (0.1 μg/ml) at 37 oC for various periods of time (specified over the lanes). Each lane was loaded with a 6,75 μg portion of digested albumin; other details as specified in the main article. The gentle digestion procedure facilitated fragmentation of large aggregates which otherwise would not enter the gel while conserving distinct patterns characteristic for BSA-Alk and BSA-AuNC. Specifically: a clear distinction between samples of BSA-Alk and BSA-AuNC digested for 5 minutes (lanes 2, and 3, respectively) is observed. The double band around 15 kDa is resolved only in trypsin-treated BSA-Alk samples. Quantitative densitometric measurements of the band’s intensity for BSA-Alk and BSA-AuNC (analyzed areas are marked with yellow windows) were carried out using ImageJ software. The obtained intensity ratio of the BSA-Alk and BSA-AuNC areas was approximately 10:1. This implies that the concentration of protein species with BSA-Alk-like trypsin-digestion characteristics does not exceed 9%. Importantly, the “BSA-Alk-like trypsin-digestion characteristics” does not equal being BSA-Alk. Although similar situation is observed for protein samples digested for 10 minutes, we note that with the digestion time increasing further, the contrast decreases due to fragmentation of larger BSA-AuNC species. (PDF) [file pone.0218975.s001.pdf]

S1 Fig. Estimation of the upper limit of residual albumin with BSA-Alk-like characteristics in BSA-AuNC samples: SDS-PAGE of partially digested BSA-Alk and BSA-AuNC.

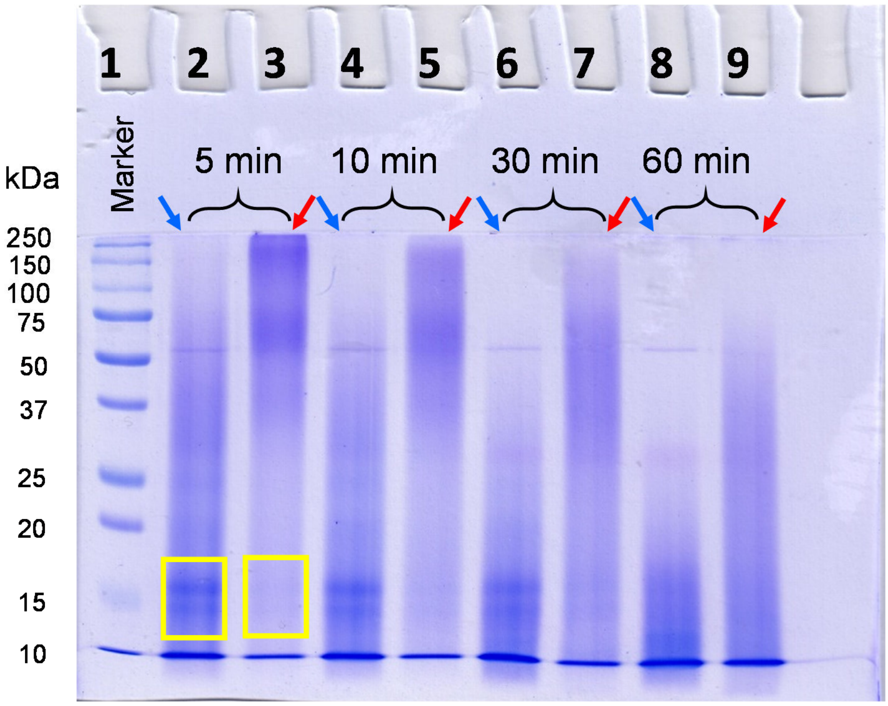

SDS-PAGE analysis of BSA-Alk (marked with blue arrows, lines 2, 4, 6, 8) and BSA-AuNC (marked with red arrows, lines 3, 5, 7, 9) gently digested with diluted trypsin (0.1 µg/ml) at 37 °C for various periods of time (specified over the lanes). Each lane was loaded with a 6,75 µg portion of digested albumin; other details as specified in the main article. The gentle digestion procedure facilitated fragmentation of large aggregates which otherwise would not enter the gel while conserving distinct patterns characteristic for BSA-Alk and BSA-AuNC.

Specifically: a clear distinction between samples of BSA-Alk and BSA-AuNC digested for 5 minutes (lanes 2, and 3, respectively) is observed. The double band around 15 kDa is resolved only in trypsin-treated BSA-Alk samples. Quantitative densitometric measurements of the band's intensity for BSA-Alk and BSA-AuNC (analyzed areas are marked with yellow windows) were carried out using ***ImageJ*** software [1,2]. The obtained intensity ratio of the BSA-Alk and BSA-AuNC areas was approximately 10:1. This implies that the concentration of protein species with *BSA-Alk-like trypsin-digestion characteristics does not exceed ~9 %.* Importantly, the "BSA-Alk-like trypsin-digestion characteristics" **does not equal being BSA-Alk**. Although similar situation is observed for protein samples digested for 10 minutes, we note that with the digestion time increasing further, the contrast decreases due to overlap with diffuse products of fragmentation of larger BSA-AuNC species.

[1] Rasband, W.S., ImageJ, U. S. National Institutes of Health, Bethesda, Maryland, USA, https://imagej.nih.gov/ij/, 1997-2018. [2] Schneider, C.A., Rasband, W.S., Eliceiri, K.W. "NIH Image to ImageJ: 25 years of image analysis". Nature Methods 9, 671-675, 2012.
