# Supplementary material for: Revisiting the conformational state of albumin conjugated to gold nanoclusters: A self-assembly pathway to giant superstructures unraveled
Source: PLoS One. 2019 Jun 27;14(6):e0218975. doi: 10.1371/journal.pone.0218975 (PMC6597083; doi:10.1371/journal.pone.0218975)
Supplement: S2 Fig — Effect of increasing concentration of GdnHCl on luminescence of BSA-AuNC excited at 365 nm (spectra collected at pH 7 and 25 oC); the cut-off peak marked with an asterisk corresponds to scattered 2*λexc light. Quantitative dependences of luminescence intensity at 640 nm and luminescence λmax on GdnHCl concentration are shown in the inset. (PDF) [file pone.0218975.s002.pdf]

S2 Fig. Titration of BSA-AuNC with GdnHCl: luminescence of AuNCs.

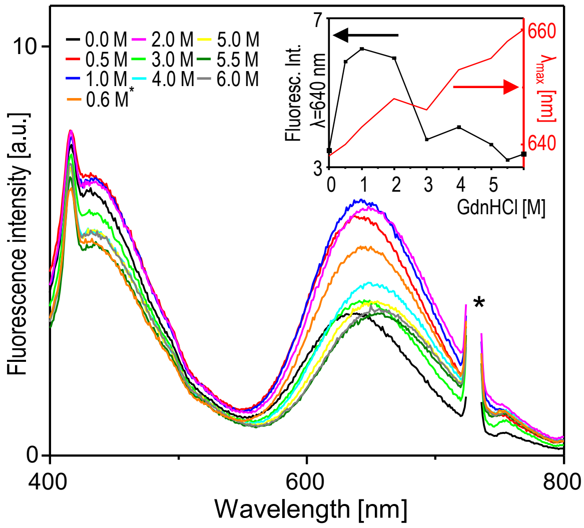

Effect of increasing concentration of GdnHCl on luminescence of BSA-AuNC excited at 365 nm (spectra collected at pH 7 and 25 ^{o}C); the cut-off peak marked with an asterisk corresponds to scattered 2*λ_{exc} light. Quantitative dependences of luminescence intensity at 640 nm and luminescence λ_{max} on GdnHCl concentration are shown in the inset.
