# Supplementary material for: Revisiting the conformational state of albumin conjugated to gold nanoclusters: A self-assembly pathway to giant superstructures unraveled
Source: PLoS One. 2019 Jun 27;14(6):e0218975. doi: 10.1371/journal.pone.0218975 (PMC6597083; doi:10.1371/journal.pone.0218975)
Supplement: S3 Fig — Experimental conditions were the same as used for SDS-PAGE analysis described in Fig 2 of the main article. Comment: Aggregate samples consisted mostly of high-molecular-weight species insoluble in SDS-PAGE buffer. However, these aggregates turned out to be sensitive to a degree to trypsin digestion. The higher concentration of trypsin used in the assay led to degradation and simultaneous release of smaller fragments of diverse sizes that revealed themselves as smears along the gel’s lanes. On the other hand significant portions of oligomers remained at the resolving gel boundary for each type of sample. The lower concentration of trypsin was ineffective in fragmentation of largest aggregates. (PDF) [file pone.0218975.s003.pdf]

S3 Fig. SDS-PAGE analysis of susceptibility to digestion by trypsin of aggregates: {BSA}, {BSA-Alk}, and {BSA-AuNC} under non-reducing conditions.

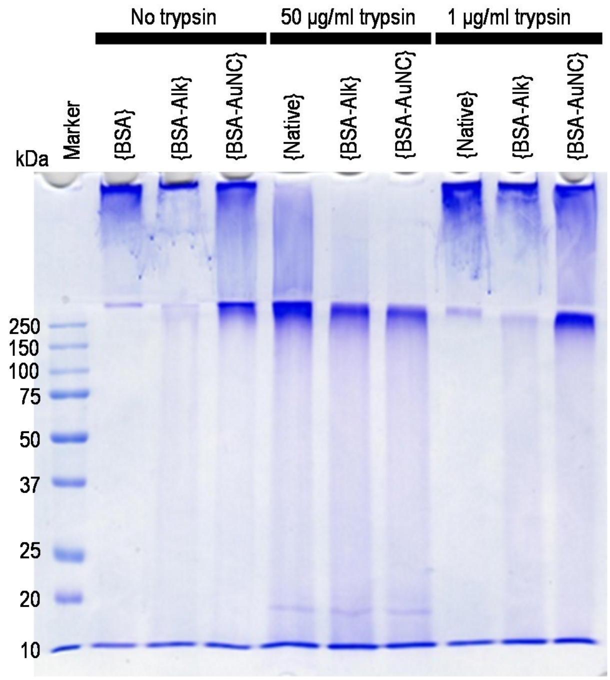

Experimental conditions were the same as used for SDS-PAGE analysis described in Fig. 2 of the main article.

Comment: Aggregate samples consisted mostly of high-molecular-weight species insoluble in SDS-PAGE buffer. However, these aggregates turned out to be sensitive to a degree to trypsin digestion. The higher concentration of trypsin used in the assay led to degradation and simultaneous release of smaller fragments of diverse sizes that revealed themselves as smears along the gel's lanes. On the other hand significant portions of oligomers remained at the resolving gel boundary for each type of sample. The lower concentration of trypsin was ineffective in fragmentation of largest aggregates.
