# Supplementary material for: Revisiting the conformational state of albumin conjugated to gold nanoclusters: A self-assembly pathway to giant superstructures unraveled
Source: PLoS One. 2019 Jun 27;14(6):e0218975. doi: 10.1371/journal.pone.0218975 (PMC6597083; doi:10.1371/journal.pone.0218975)
Supplement: S4 Fig — Aggregates were observed using optical microscopy (A) and SEM (B-D). The fractal-like arrangements of aggregates were also observed using tapping mode AFM on mica support as shown in (E). Cross-sections of selected specimen of aggregates obtained from the corresponding AFM height images are displayed in panel (F). Comment: The large size of aggregates enabled detecting them even using optical microscopy: panel (A). The higher resolution afforded by SEM confirmed the presence of triangular forms (B-C) also in the case of aggregates deposited on silicon wafer (instead of Formvar/carbon substrates used for TEM imaging). The fractal arrangements of {BSA-AuNC} were also observed in SEM and AFM images vide amplitude AFM image in panel (E) collected on a mica substrate, although the latter technique is best-suited for flat aggregates that do not vary greatly in depth, as {BSA-AuNC} tend to do. When the acquisition of proper AFM images was possible, cross-sections were obtained from the corresponding height images (F). Such sufficiently flat specimen were estimated to be on average 10–25 nm thick, i.e. remarkably more than the diameter of typical amyloid fibrils. (PDF) [file pone.0218975.s004.pdf]

S4 Fig. Morphology of {BSA-AuNC}.

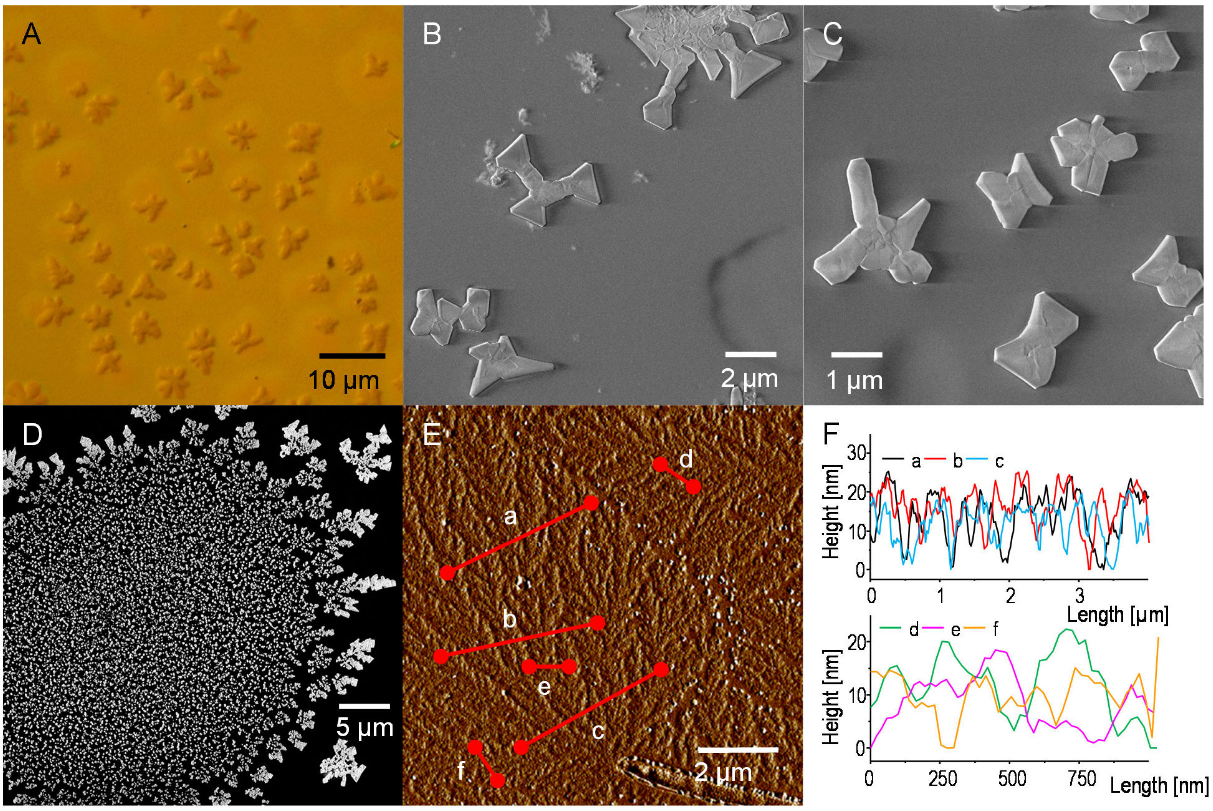

Aggregates were observed using optical microscopy (A) and SEM (B-D). The fractal-like arrangements of aggregates were also observed using tapping mode AFM on mica support as shown in (E). Cross-sections of selected specimen of aggregates obtained from the corresponding AFM height images are displayed in panel (F).

Comment: The large size of aggregates enabled detecting them even using optical microscopy : panel (A). The higher resolution afforded by SEM confirmed the presence of triangular forms (B-C) also in the case of aggregates deposited on silicon wafer (instead of Formvar/carbon substrates used for TEM imaging). The fractal arrangements of {BSA-AuNC} were also observed in SEM and AFM images vide amplitude AFM image in panel (E) collected on a mica substrate, although the latter technique is best-suited for flat aggregates that do not vary greatly in depth, as {BSA-AuNC} tend to do. When the acquisition of proper AFM images was possible, cross-sections were obtained from the corresponding height images (F). Such sufficiently flat specimen were estimated to be on average 10-25 nm thick, i.e. remarkably more than the diameter of typical amyloid fibrils.
