# Supplementary material for: Revisiting the conformational state of albumin conjugated to gold nanoclusters: A self-assembly pathway to giant superstructures unraveled
Source: PLoS One. 2019 Jun 27;14(6):e0218975. doi: 10.1371/journal.pone.0218975 (PMC6597083; doi:10.1371/journal.pone.0218975)
Supplement: S5 Fig — Control TEM images of {BSA-AuNC} subjected to preliminary 24h-long dialysis against 100-fold excess of deionized water (A-B). The key morphological features of the aggregates including twisted superstructures and diffuse early aggregates are clearly preserved when traces of salts are removed. (C) TEM image of {BSA-Alk}. Comment: Interestingly, higher abundance of the diffuse forms in dialyzed aggregates suggests that the kinetics of maturation of {BSA-AuNC} may strongly depend on minute variations in ionic strength. The presence of clumped entities in {BSA-Alk} (C) suggests that the perturbation in BSA caused by the alkaline treatment may be a key factor predisposing the protein envelope to self-associate which clearly plays a significant role in the self-assembly pathway of {BSA-AuNC}. (PDF) [file pone.0218975.s005.pdf]

S5 Fig. Control data: morphology of {BSA-AuNC} after removal of salts through dialysis, morphology of {BSA-Alk}.

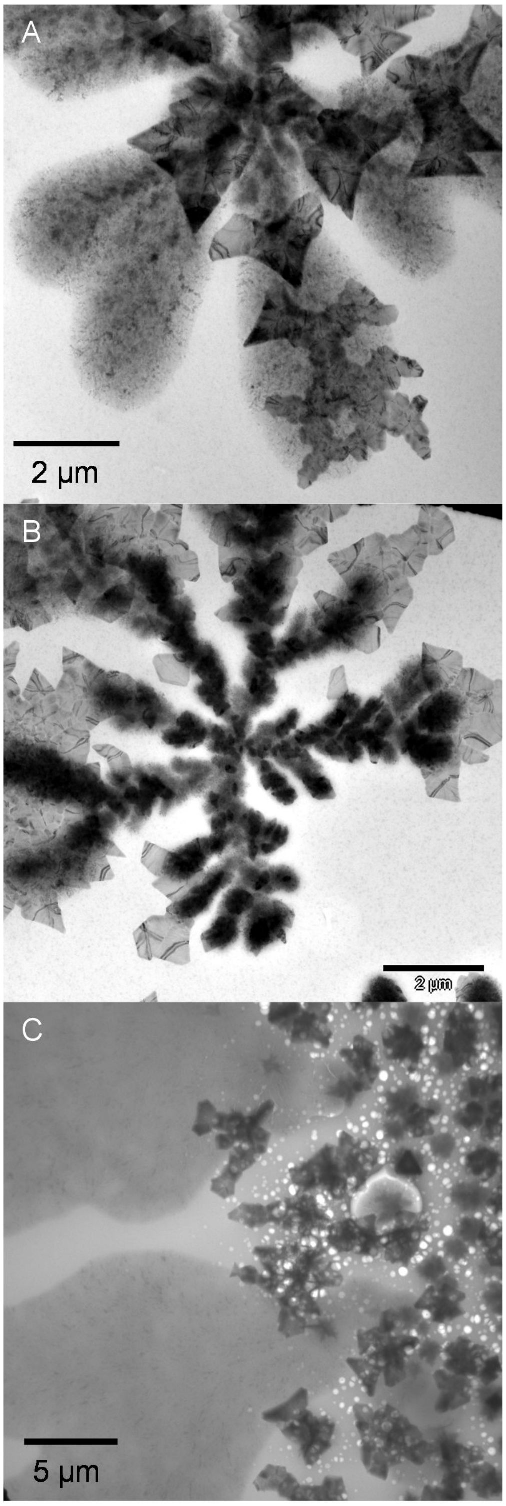

Control TEM images of {BSA-AuNC} subjected to preliminary 24h-long dialysis against 100-fold excess of deionized water (A-B). The key morphological features of the aggregates including twisted superstructures and diffuse early aggregates are clearly preserved when traces of salts are removed. (C) TEM image of {BSA-Alk}.

Comment: Interestingly, higher abundance of the diffuse forms in dialyzed aggregates suggests that the kinetics of maturation of {BSA-AuNC} may strongly depend on minute variations in ionic strength. The presence of clumped entities in {BSA-Alk} (C) suggests that the perturbation in BSA caused by the alkaline treatment may be a key factor predisposing the protein envelope to self-associate which clearly plays a significant role in the self-assembly pathway of {BSA-AuNC}.
